# Acceptability of an Alimentary Supplement of Whey-Protein Concentrate and TGF-****β**** in Patients with Crohn's Disease

**DOI:** 10.5402/2013/947865

**Published:** 2013-07-21

**Authors:** Taciana Davanço, Luciano Bruno de Carvalho Silva, Karina de Lemos Sampaio, Cláudio Saddy Rodrigues Coy, Maria Marluce dos Santos Vilela, Elizete Aparecida Lomazi da Costa Pinto

**Affiliations:** ^1^Nutrition Department, Padre Anchieta University Center, 13210-800 Jundiaí, SP, Brazil; ^2^Faculty of Nutrition, Federal University of Alfenas, 37130-000 Alfenas, MG, Brazil; ^3^Food and Nutrition Department, Faculty of Food Engineering, University of Campinas (UNICAMP), 13083-862 Campinas, SP, Brazil; ^4^Faculty of Medical Sciences, University of Campinas, 13083-887 Campinas, SP, Brazil

## Abstract

The objective of this study was to evaluate the acceptability of an alimentary supplement of bovine whey-protein concentrate (WPC) and TGF-**β**, unavailable commercially, by patients with Crohn's disease (CD) and determine the chemical composition, solubility, and total amino acids content. The supplement was diluted in water, and an acceptance test was done to evaluate the aroma, flavour, and viscosity of the product using facial hedonic scale (nine-point scale), applied on 54 CD patients. The supplement composition indicated 73.3% protein, 10.5% fat, 2.2% ash, 6.3% water, and 7.7% carbohydrate. The supplement is presented as a good protein source and high content of essential amino acids. The average acceptance for all the attributes was between 5.0 and 6.0, and the flavour was mainly associated with soybean/grain, sour milk, and sweet/vanilla flavour. The results indicated that the supplement provided important nutritional properties for CD patients; however, for a large number of individuals to be encouraged to perform supplementation, it is essential to improve the sensory quality of the product. In order to do so, additional research is necessary to prevent the formation of volatiles which cause off-flavours or to mask undesirable aromas/flavours found in it.

## 1. Introduction 

The interest in whey-protein supplement (WPS) increased with consumer's sensibility concerning health benefits, since the immunomodulatory role of WPS has already been amply demonstrated [1, 2]. The whey protein (WP) constitutes 20% of the total protein content of bovine milk and includes alpha-lactalbumin (*α*-LA), beta-lactoglobulin (*β*-LG), bovine serum albumin (BSA), lactoferrin (LTF), immunoglobulins (Ig), and growth factors to tissues. They are associated with the prebiotic effect, promoting tissue restoration, maintenance of intestinal integrity, destruction of pathogens, and elimination of toxins [3]. Highly digestible and quickly absorbed by the body, the WPs are widely used in situations of metabolic stress [4]. Whey-protein concentrates (WPCs) and whey-protein isolates (WPIs) usually contain a cysteine concentration at least four times higher than other high-quality proteins, and it produces an improvement in immune response [5]. 

Crohn's disease (CD) is a complex chronic inflammatory disease, with periods of exacerbation [6], which affects the gastrointestinal tract, and may also involve the musculoskeletal system, skin, and eyes [7]. It is important to consider that although the CD may affect any portion of the digestive tract, in most cases it occurs more frequently in the small intestine. The ileum and cecum are affected in 40% of cases, the small intestine in 30%, and colon in 25% [8]. With this large percentage of presentation in the small intestine, the disease can be extremely harmful to the nutritional status of patients.

The deregulation of the immune system associated with the intestinal mucosa leads to chronic inflammation, which leads to the destruction of intestinal epithelium and severe functional abnormalities, with loss of absorptive capacity and excessive secretion of electrolytes and intestinal fluids. Thus, CD individuals may show nutritional changes ranging from deficiency of trace elements to severe malnutrition [9]. Several types of diets and supplements are able to modulate the immune system functions, and their bioactive components have been proposed to form the basis of functional immunomodulatory food product [10]. Human and bovine milk contains the transforming growth factor *β* (TGF-*β*), multifunctional polypeptide with an important role in the development of tolerance and prevention of autoimmunity [11]. Despite the valuable functional and nutritional properties, it is important to note that WP has little or no flavour [12]. However, some compounds in whey are susceptible to chemical reactions, for example, lipid oxidation forming unpleasant aromas and flavours in the product [13, 14]. Today, consumers increasingly expect to obtain pleasure from food and require sensory characteristics, for example, aroma, flavour, texture, and, along with these sensory characteristics, maintaining or improving their health and welfare. Therefore, for CD patients (who often require specific nutritional support) to be encouraged to undertake the process of continuous alimentary supplementation, it is necessary to study the acceptability of the product, so that researchers and industries can adapt to the sensory quality of the product as expected by patients. 

Thus, the objective of this study was to evaluate the acceptability of an alimentary supplement of whey-protein concentrate (WPC) and TGF-*β*, unavailable commercially, by patients with Crohn's disease. It is the first time that a study about the acceptability of this supplement by CD patients is done. In this study the chemical composition, solubility and total amino acids content were also determined.

## 2. Material and Methods

### 2.1. Material

The supplement used was donated by an industry of cheese and whey products from California (USA). The supplement was constituted by whey-protein concentrate (WPC) with the addition of TGF-*β* in the exogenous shape, but in amounts not reported by the company, since it is a supplement not marketed yet. 

### 2.2. Chemical Composition of the Supplement

The humidity, total solids, ash, and protein were established based on the methods of AOAC [15]. Total lipids were determined according to Bligh and Dyer [16] and total carbohydrates were estimated by difference, subtracting the sum of the values obtained in other measurements of 100%.

### 2.3. Protein Solubility

The water protein solubility (%) was determined according to the method of Morr and Foegeding [17]. The effects of different pH (from 2.5 to 7.5) were also evaluated.

### 2.4. Total Amino Acid Determination

Total amino acids in the supplement were determined by reversed phase liquid chromatography, after acid hydrolysis (24 h), plus 20% of HCl phenol, followed by derivatisation with phenylisothiocyanate [18].

### 2.5. Recruiting and Selecting Individuals

Fifty-four individuals (24 male and 30 female) who had CD and aged from 18 to 62 years (median age of 37 years) were invited to participate in this study. It was conducted in the Gastrocentro (Centre for Diagnosis of Digestive Tract Diseases), University of Campinas (UNICAMP), Brazil. The patients with CD performed the acceptance test of the supplement in a single session for the three attributes: aroma, flavour, and viscosity, according to the following criteria: (a) patients of both genders; (b) attending outpatient department regularly. 

This study was approved by the local ethics committee under protocol 304/2007, and all individuals signed an informed consent form before the sensory evaluation.

### 2.6. Preparing the Samples

The patients conducted an acceptance test assessing the supplement in Inflammatory Intestinal Disease Outpatient Clinic at Gastrocentro (UNICAMP). Such test was previously prepared by diluting 15 g of the product in 100 mL of filtered water at room temperature. The test was performed in individual booths and during the morning from 9 : 00 to 11 : 00 h. 

### 2.7. Sensory Analysis

**Figure 1:**
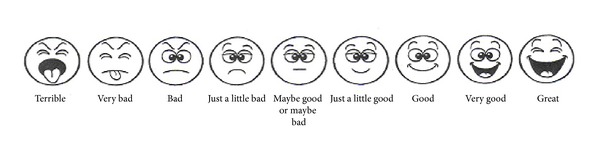
Facial hedonic scale used in the present study [19].

The CD patients assessed in a single session the acceptance of the product concerning the attributes: aroma, flavor, and viscosity by using nine-point facial hedonic scale in the extreme left and extreme right, under the term “terrible” and the term “great,” as shown in Figure 1 [19, 20]. 

To evaluate the acceptance results, the items of hedonic facial scale were converted into numerical values. Thus, the term “terrible” was converted into the number 1, the term “very bad” was converted into the number 2, the term “bad” into the number 3, and so on, until the term “great,” that was converted to number 9 in the scale.

For aroma and flavour, the patients were also asked to quote the aromatic notes and flavours perceived in the supplement sample and to assess the product purchase intention.

### 2.8. Statistical Analysis

The physicochemical data were assessed by calculating the average, standard deviation, and Tukey's test, by using the software SPSS 15.1 for Windows [21]. Sensory analysis data were assessed by calculating average, and perceived percentage of quotes for aroma and flavours, processed in the Microsoft Excel 2002.

## 3. Results and Discussion

### 3.1. Physicochemical Characterization of The Supplement

Table 1 shows the supplement chemical composition used in this study. In its composition, the supplement had high protein value and low carbohydrate content.

Due to the high content of essential amino acids, especially the branched chain ones, the supplement also showed high nutritional value, as noted in Table 2. These values are above average when compared to those from other protein sources, for example, casein, soybean, egg, rice, beans, chicken, and so forth, providing supplement with important nutritional properties. It may contribute to maintain the nutritional status for CD patients, who often require special nutritional support.

Due to the WPC amino acids profile, this protein is used to formulate several specialty products, for example, infantile formulas, and for the performance of muscle metabolism, due to the high content of essential amino acids branched chain, for example, leucine and isoleucine [22]. These characteristics are hugely important for CD patients, due to hypermetabolism and progressive loss of lean mass, with the clinical evolution of the disease.

The results indicated that the supplement has good solubility in a wide pH range (Table 3), which is quite advantageous, since it may be used to formulate various foods, for example, fermented beverages, ice creams, and so forth [23]. Thus, it increases the possibilities for consumption of this supplement with high nutritional value.

### 3.2. Sensory Evaluation

According to the acceptance averages for aroma, flavour, and viscosity obtained for the CD patients, it was observed that the assessment of the supplement in general was between the terms “maybe good or maybe bad” and “just a little good” (Table 4). 

**Figure 2:**
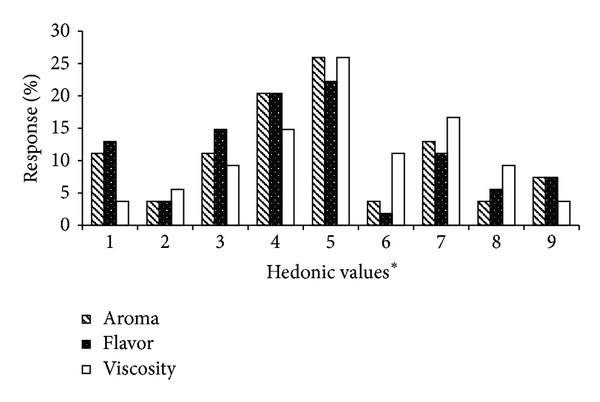
Distributing patients according to the hedonic values to accept the aroma, flavour, and viscosity, provided to the supplement. Hedonic values* 1 = terrible, 5 = maybe good or maybe bad, and 9 = great.

According to the frequency of responses to aroma, 46.3% of values were in the region of product rejection (values from 1 to 4 or between the words “terrible” and “just a little bad”). By contrast, 25.9% of the assessed individuals classified the supplement as “maybe good or maybe bad” and 28% of patients assessed the supplement between “just a little good” and “great” (values between 6 and 9 on the scale) (Figure 2).

In fact, the supplement aroma was characterised by presenting the unpleasant aroma notes in percentage of responses, such as soybean/grain (31%), sour milk (17%), and cooked food (9%). It was observed that a small proportion of quotes were associated with pleasant aromas, for example, sweet/vanilla (17%). Concerning the flavour, the frequency of responses was quite similar to the aroma: nearly 28% of patients liked the product and 50% did not like it. Additionally, the flavour was mainly associated with soybean/grain flavour (34% of respondents), sour milk (16% of respondents), sweet/vanilla (13%), and milk powder (9%) (Table 5). For viscosity, the acceptance was a little better; nearly 40% liked the sample viscosity against 33% who did not like it.

In general, it was observed that roughly 30% of individuals liked the supplement concerning all attributes assessed. By contrast, half of them did not like it and the other, from 20% to 25%, classified it as “maybe good or maybe bad.” It indicates that the product needs to be improved, especially its aroma and flavour. On the other hand, the acceptance was not as bad as the supplement was evaluated only dissolved in water, maybe if the supplement was added to fruit juice or milk drink could have a better acceptance by the patients.

The aroma and flavour of grain/soybean, sour milk, bitter, cheese, cooked food, all noticed by individuals, can be from lipid oxidation or Maillard reaction (or both), as some studies have shown. These reactions contribute to the formation of off-flavours and to the loss of pleasant aromas/flavours of whey-protein concentrate, thus limiting its use [24]. Quach et al. [25] by headspace-SPME (solid-phase microextraction) isolated and identified 43 volatile from WPC, among them several aldehydes, ketones, and hydrocarbons. According to the authors, several of them can contribute to the aromas and flavours perceived in WPC, for example, 2-heptanone, 2-nonanone, 1-octen-3-ol, and volatiles associated with aromas of cardboard, metal, and mould, respectively. 

The formation of volatiles during production and storage of WPC is difficult to control, considering all the reactions that may occur. To obtain a WPC with milder aroma/flavour would be necessary to standardise the conditions for processing and storage of WPC, and thus obtain a supplement with better sensory quality.

Despite the low acceptance of the product, 28% of patients indicated that they “probably would buy” the supplement, and 22% said they “definitely would buy” the product. Such fact indicates a good purchase intention. By contrast, 19% of patients indicated that they “perhaps would or would not buy” the supplement, 22% said they “probably would not buy” the product, and 9% of them “definitely would not buy”. 

Several studies have demonstrated that complete nutrition with protein supplements improves nutritional status of patients, intestinal inflammation, and, often, recovery of intestinal mucosa [26]. Paradoxically, the treatment using only corticosteroids presents limited effects on the mucosa [27]. In children affected by CD, complete nutrition plays a fundamental role in the increase of their growth, since the additional nutrient provision is coupled with improvement in intestinal inflammation [26]. In CD-acute adults, the complete nutrition is greatly effective in relieving the disease and in the nutritional status of individuals, and it can be used together to other therapies to control CD [28]. Therefore, studies assessing and developing oral solutions that are better accepted by patients are very important.

## 4. Conclusion

The supplement presented as a good protein source and high content of essential amino acids providing important nutritional properties that can contribute to maintain the nutritional status for CD patients, who often require special nutritional support.

Although the supplement was not well accepted for aroma, flavour, and viscosity by most patients, almost 30% of individuals liked the supplement concerning all attributes assessed. The results indicated that the supplement provided important nutritional properties for patients with CD; however, for a large number of patients to be encouraged to start the supplementation process, it is essential to improve the sensory quality of the product. 

In order to do so, additional research is necessary to prevent the formation of volatiles, which cause off-flavours in WPC, associated in the present study with soybean/grain, sour milk, cooked food, vomit, and other undesirable aromas and flavours. 

## Figures and Tables

**Table 1:** Chemical composition of the supplement.

Protein (%)^1,2^	Fat (%)^1,2^	Ash (%)^1,2^	Water (%)^1,2^	Carbohydrate (%)^2,3^
73.30 ± 0.43	10.48 ± 0.03	2.15 ± 0.33	6.36 ± 0.01	7.71 ± 0.01

^1^Values according to averages (±SD) of three determinations. ^2^Values expressed on dry basis.

^3^Calculated by difference = 100 − (total protein + fat + ash + water).

**Table 2:** Total amino acids content in the supplement.

Amino Acids	(g/100 g of protein)
Aspartic acid	10.16
Glutamic acid	16.95
Serina	5.18
Glycine	2.00
Histidine	1.79
Arginine	1.44
Threonine	6.22
Alanine	5.00
Proline	5.90
Phenylalanine and tyrosine	5.47
Valine	5.64
Methionine and cystine	3.10
Cysteine	2.01
Isoleucine	6.49
Leucine	10.16
Lysine	9.32
Tryptophan	*

*nondetermined.

**Table 3:** Solubility of the supplement in water.

pH	Supplement solubility (%)^1,2^
2.5	71.56 ± 1.45^C^
3.5	80.93 ± 0.53^A^
4.5	77.54 ± 0.11^B^
5.5	80.71 ± 0.28^A^
6.5	81.18 ± 0.56^A^
7.5	79.88 ± 0.72^A^

^1^Values for averages (±SD) of three determinations.

^2^Values with different letters mean significant difference, according to Tukey's test (*P* < 0.05).

**Table 4:** Acceptance averages of aroma, flavour, and viscosity (*n* = 54 patients).

Attributes	Acceptance*
Aroma	5.3
Flavour	5.5
Viscosity	5.5

*Values on the scale: 1 = terrible; 5 = maybe good or maybe bad; 9 = great.

**Table 5:** Citation frequency (%) of perceived aroma notes and perceived flavours by individuals in the supplement (*n* = 54 patients).

Descriptor	Aroma (%)	Flavour (%)
Grain/soybean	31	34
Sour milk	17	16
Sweet/vanilla	17	13
Cooked food	9	7
Milk powder	8	9
Cheese	8	1
Vomit	4	7
Metallic	4	6
Earth	2	4
Bitter	0	3
